# *pfk13*-Independent Treatment Failure in Four Imported Cases of Plasmodium falciparum Malaria Treated with Artemether-Lumefantrine in the United Kingdom

**DOI:** 10.1128/AAC.02382-16

**Published:** 2017-02-23

**Authors:** Colin J. Sutherland, Paul Lansdell, Mandy Sanders, Julian Muwanguzi, Donelly A. van Schalkwyk, Harparkash Kaur, Debbie Nolder, Julie Tucker, Hayley M. Bennett, Thomas D. Otto, Matthew Berriman, Trupti A. Patel, Roderick Lynn, Effrossyni Gkrania-Klotsas, Peter L. Chiodini

**Affiliations:** a Public Health England Malaria Reference Laboratory, London School of Hygiene & Tropical Medicine, London, United Kingdom; b Department of Immunology & Infection, Faculty of Infectious & Tropical Diseases, London School of Hygiene & Tropical Medicine, London, United Kingdom; c Wellcome Trust Sanger Institute, Hinxton, United Kingdom; d Hospital for Tropical Diseases, London, United Kingdom; e Haematology Department, Addenbrooke's Hospital, Cambridge University Hospitals NHS Foundation Trust, Cambridge, United Kingdom; f Infectious Diseases Department, Addenbrooke's Hospital, Cambridge University Hospitals NHS Foundation Trust, Cambridge, United Kingdom

**Keywords:** imported malaria, treatment failure, parasite genotyping, antimalarial agents, drug resistance mechanisms

## Abstract

We present case histories of four patients treated with artemether-lumefantrine for falciparum malaria in UK hospitals in 2015 to 2016. Each subsequently presented with recurrent symptoms and Plasmodium falciparum parasitemia within 6 weeks of treatment with no intervening travel to countries where malaria is endemic. Parasite isolates, all of African origin, harbored variants at some candidate resistance loci. No evidence of *pfk13*-mediated artemisinin resistance was found. Vigilance for signs of unsatisfactory antimalarial efficacy among imported cases of malaria is recommended.

## TEXT

Treatment of Plasmodium falciparum malaria has benefited from the availability of artemisinin-based combination therapies (ACT) (1). In Asia, reduced artemisinin susceptibility mediated by specific mutations in the parasite *pfk13* locus may indicate a threat to ACT efficacy (2, 3). However, these specific *pfk13* variants have not been seen in Africa (2–4), where variable ACT efficacy appears to be independent of *pfk13* polymorphism but may be linked to multilocus genotypes encompassing *pfcrt*, *pfmdr1*, *pfap2mu*, and *pfubp1* (5–9).

Artemether-lumefantrine (AL) is the recommended treatment for imported cases of falciparum malaria in the UK (1) and is the most widely used ACT in Africa, the origin of most UK infections (10). Between October 2015 and February 2016, four cases of recurrent P. falciparum malaria in AL-treated patients occurring 17 to 43 days after the original episode were reported to the Malaria Reference Laboratory (MRL) (Table 1) by two UK hospitals. Brief case reports and genotyping of parasites isolated from both episodes in each patient are presented.

**TABLE 1:** Genotyping data from P. falciparum isolates taken from primary and secondary symptomatic malaria episodes in four UK patients

Patient	Sex and age (yrs)	Travel	Episode 1	Episode 2 (elapsed time since previous episode)	Mutation(s) or haplotype					*WGS*^*f*^ clonality
					*pfk13*^*b*^	*pfcrt*^*c*^	*pfmdr1*	*pfap2mu*	*pfubp1*	
1	Male over 65	Angola	August 2015		Lys189Thr	CVIET	Tyr184Phe^*d*^	Ser160Asn	WT	
				43 days	Lys189Thr	CVIET	Tyr184Phe	Ser160Asn	WT	
2	Female under 21	Uganda	January 2016		WT	CVIET	WT	WT	Glu1525Gln	
				28 days (cultured)^*a*^	WT	CVIET	WT	WT	Glu1525Gln	
3	Male over 21	Liberia	February 2016		WT Gly112Glu	CVIET CVMNK	Tyr184Phe	ins_Lys233,^*e*^ ins_Asn233	WT	≥2 clones
				17 days	Gly112Glu	CVMNK	Tyr184Phe	ins_Lys233	WT	1 clone
4	Female under 21	Uganda	January 2016		Lys189Thr	CVIET	WT	ins_Asn226, ins_Asn326	Thr1500Ile	
				34 days	Lys189Thr	CVIET	WT	ins_Asn226, ins_Asn326	Thr1500Ile	

a Parasites from the second malaria episode of patient 2 were successfully established in *in vitro* culture for estimation of drug susceptibility.

b The nomenclature used shows the wild-type (WT) (reference) amino acid encoded at the codon of interest, followed by the amino acid encoded by the observed variant. Mutations in the *pfk13* locus previously associated with reduced susceptibility to artemisinin *in vitro* and *in vivo* in the Mekong region occur between codon 440 and the 3′ end of the coding region.

c The nomenclature used shows the single-letter amino acid code for codons 72 to 76, strongly associated with chloroquine resistance. The CVMNK haplotype has been associated with AL treatment failures in Kenya (5).

d The single mutation Tyr to Phe at codon 184 identifies the NFD genotype at codons 86, 184, and 1246 in the *pfmdr1* locus, previously associated with AL failure in Tanzania and Kenya (5, 8).

e ins, insertion of one amino acid, relative to the reference sequence, after the codon shown.

f Whole-genome sequencing results for both parasite isolates from patient 3. In the primary episode, heterozygosity (variant allele frequencies of between 5% and 95% of total reads) was seen in over 4,000 loci. About 50% of reads at informative positions comprised variants also present in the monoclonal recrudescent episode (17 days later).

This work was undertaken under the statutory remit of the MRL set out by the UK Department of Health, which explicitly requires surveillance for evidence of emerging drug resistance among UK malaria cases, as previously described (10). All tests performed were formally requested by physicians directly responsible for patient care. All patient identifiers were removed from the data prior to extraction from the Public Health England (PHE) MRL database. Other than those required for the primary diagnosis of malaria, no additional blood samples were taken from patients. Patient 2 gave written informed consent for culture adaption of parasites from her blood samples, under UK National Research Ethics Service Approval 07/Q0505/60, granted to C. J. Sutherland.

### Patient 1.

A male over 65 years of age presented to Addenbrooke's Hospital, Cambridge University Hospital (CUH), UK, in 2015 with 1.4% P. falciparum parasitemia following travel in Angola. He was treated with AL in the hospital, and all doses were observed. The first dose was consumed without food, and the patient vomited once 3 h later. All but one of the subsequent AL doses were consumed with food, and the patient was confirmed negative for parasitemia prior to discharge. Symptoms recurred more than 1 month later, and the patient presented with 0.02% P. falciparum parasitemia, confirmed by PCR and microscopy at the MRL. The patient responded well to administration of 4 tablets of 100/250 mg atovaquone/proguanil daily for 3 days. The *pfmdr1* Tyr184Phe and *pfap2mu* Ser160Asn variants observed have both been associated with recurrent parasitemia in AL-treated African patients (5, 8).

### Patient 2.

A Ugandan female under 21 years of age was admitted to the Hospital for Tropical Diseases, London (HTD), with acute P. falciparum hyperparasitemia estimated at more than 30% in early 2016 following a visit to Uganda. She responded well to initial treatment with intravenous (i.v.) artesunate. Parasitemia of 0.07% was recorded 60 h after admission, and the patient was discharged 3 days later, with a recorded parasitemia level of 0.0001%, on a full course of oral AL. Full compliance was reported under supervision of staff members at her place of learning. Symptoms recurred 4 weeks later. The patient was readmitted to HTD with 2.7% P. falciparum parasitemia, received 2 days i.v. artesunate as an inpatient until the parasitemia level fell below 0.0001%, and was discharged on a full course of AL plus 7 days of doxycycline (200 mg daily). This recurrent isolate was established in *in vitro* culture at the London School of Hygiene & Tropical Medicine (LSHTM), and standard 48-h 50% effective concentration (EC_50_) estimates were derived for chloroquine (102.6 nM), dihydroartemisinin (2.8 nM), and lumefantrine (38.7 nM) as previously described (11), with the results indicating full sensitivity to AL components *in vitro*. The genotypes of both parasite isolates were consistent with AL sensitivity at *pfk13*, *pfcrt*, *pfmdr1*, and *pfap2mu* (Table 1). However, the Glu-to-Gln change at codon 1525 of *pfubp1* was associated with reduced susceptibility to ACT *in vivo* in one study (8). The recurrent parasitemia occurring 28 days after first presentation may reflect incomplete clearance of the original hyperparasitemia by the treatment received.

### Patient 3.

A male over 21 years of age presented to CUH with 0.4% P. falciparum parasitemia in early 2016, after travel to Liberia. He was treated with AL and was eventually discharged but returned with recurrent symptoms and 0.02% parasitemia 17 days later. He was successfully treated with atovaquone-proguanil. Genotyping identified mixed genotypes at both *pfcrt* and *pfap2mu* loci in the isolate from the primary episode but not in that from the recurrent episode (Table 1). DNA extracted from the patient after both episodes was sent to the Wellcome Trust Sanger Institute for whole-genome sequencing (WGS). Preliminary analysis strongly suggests that the recurrent infection which occurred 17 days after AL treatment comprised a single parasite clone, whereas the original parasite population was multiclonal (Table 1). The recurrent isolate harbored the previously identified CVMNK and NFD haplotypes of *pfcrt* and *pfmdr1*, known to be selected in parasites persisting after AL treatment (5, 8). The impact of the ins_Lys233 variant of *pfap2mu* and that of the novel *pfk13* non-propeller region mutation Gly112Glu require further study. Analysis in the bioanalytical laboratory of LSHTM confirmed that the AL tablets used to treat this patient (Novartis Riamet [lot XO131; expiration date, December 2017]) met the specified International Pharmacopeia tolerance limits (12).

### Patient 4.

A female under 21 years of age who presented as febrile to the HTD in early 2016 following travel to Uganda was diagnosed with 0.08% P. falciparum parasitemia and treated with oral AL. The patient was discharged to complete the treatment course at home and reported full compliance with all six doses. She experienced recurrent symptoms 1 month later, presenting to HTD with confirmed 0.3% P. falciparum parasitemia, and was successfully treated with 7 days of oral quinine (10 mg/kg of body weight every 8 h) and doxycycline (200 mg daily). Data from the two episodes appeared identical at the parasite loci evaluated. Novel mutations at codons 226 and 326 of *pfap2mu* and codon 1500 of *pfubp1* are of unknown significance, but, as this is clearly a recrudescent parasite genotype, both variants should be further evaluated in future studies.

Treatment failure cannot be unequivocally ascribed to parasite resistance in these four patients, although three harbored parasites with variant alleles of loci previously linked to reduced susceptibility to artemisinin or lumefantrine (Table 1). Our findings would have been strengthened by observing treatment for all patients to ensure full regimen compliance, and by measurement of lumefantrine blood levels at day 7, to rule out any problems with malabsorption (1), as this can result in failure to clear parasites (13). None of the four patients harbored mutations in the propeller-encoding domain of the *pfk13* locus and thus did not exhibit reduced artemisinin susceptibility as seen in the Greater Mekong Region (2–4). The parasites that we describe may represent adaptive African genotypes that have evolved *pfk13*-independent mechanisms for posttreatment survival *in vivo*. This suggests that, in countries where malaria is not endemic and in which imported malaria cases from Africa are seen, vigilance is required to protect the efficacy of our first-line ACT. Studies in countries where malaria is endemic are required to further validate potential genetic markers for monitoring in African parasite populations.

Artemisinins are very quickly metabolized and cleared from circulation, such that only a single full parasite life cycle is exposed to the highly potent action of these compounds under conditions of a 3-day regimen. The current practice of 3 days of treatment may therefore be insufficient to guarantee clearance of parasite genotypes under ACT pressure for a decade in many parts of Africa (14). The use of two sequential rounds of ACT, such as AL followed by 3 days of either artesunate-amodiaquine or dihydroartemisinin-piperaquine, would provide 6 days of treatment with artemisinin with two longer-half-life partner drugs (14, 15). This approach should also be considered a potential strategy should increasing numbers of treatment failures be observed after administration of standard ACT regimens for imported malaria cases in countries where malaria is not endemic. In the majority of cases, ACT retains its efficacy against P. falciparum of African origin, but the cases presented here suggest that this favorable situation may deteriorate in the future.
